# The association of depression and all-cause and cause-specific mortality: an umbrella review of systematic reviews and meta-analyses

**DOI:** 10.1186/s12916-018-1101-z

**Published:** 2018-07-20

**Authors:** Myrela O. Machado, Nicola Veronese, Marcos Sanches, Brendon Stubbs, Ai Koyanagi, Trevor Thompson, Ioanna Tzoulaki, Marco Solmi, Davy Vancampfort, Felipe B. Schuch, Michael Maes, Giovanni A. Fava, John P. A. Ioannidis, André F. Carvalho

**Affiliations:** 1 Department of Clinical Medicine and Translational Psychiatry Research Group, Faculty of Medicine, Federal University of Ceará, Fortaleza, CE 60430-140 Brazil; 2 Institute for Clinical Research and Education in Medicine (IREM), 35128 Padova, Italy; 3 National Research Council, Neuroscience Institute, Aging Branch, 35128 Padova, Italy; 4 Biostatistical Consulting Unit, Centre for Addiction and Mental Health (CAMH), Toronto, ON Canada; 5 South London and Maudsley NHS Foundation Trust, Denmark Hill, London, SE5 8AZ UK; 6 Institute of Psychiatry, Psychology and Neuroscience (IoPPN), King’s College London, De Crespigny Park, London, AF SE5 8 UK; 7 Faculty of Health, Social Care and Education, Anglia Ruskin University, Chelmsford, CM1 1SQ UK; 8 Parc Sanitari Sant Joan de Déu, Universitat de Barcelona, Fundació Sant Joan de Déu/CIBERSAM, 08950 Barcelona, Spain; 9 Faculty of Education and Health, University of Greenwich, London, SE10 9LS UK; 10 Department of Epidemiology and Biostatistics, School of Public Health, Imperial College London, W2 1PG, London, UK; 11 MRC-PHE Centre for Environment, School of Public Health, Imperial College London, London, W2 1PG UK; 12 Department of Hygiene and Epidemiology, University of Ioannina Medical School, Ioannina, Greece; 13 Department of Neuroscience, University of Padova, 35100 Padova, Italy; 14 Department of Rehabilitation Sciences, KU Leuven - University of Leuven, 3001 Leuven, Belgium; 15 KU Leuven - University of Leuven, University Psychiatric Center KU Leuven, 3070 Leuven, Kortenberg Belgium; 16 Centro Universitário La Salle, Canoas, Brazil; 17 Hospital de Clínicas de Porto Alegre, Porto Alegre, Brazil; 18 Department of Psychiatry, Faculty of Medicine, Chulalongkorn University, Bangkok, 10330 Thailand; 19 IMPACT Strategic Research Center, Barwon Health, Deakin University, Geelong, VIC Australia; 20 Department of Psychology, University of Bologna, viale Berti Pichat 5, 40127 Bologna, Italy; 21 Department of Psychiatry, Erie County Medical Center, 462 Grider Street, Buffalo, NY 14215 USA; 22 Department of Medicine, Stanford University, Palo Alto, CA 94305 USA; 23 Department of Health Research and Policy, Stanford University, Palo Alto, CA 94305 USA; 24 Department of Statistics, Stanford University, Palo Alto, CA 94305 USA; 25 Department of Meta-Research Innovation Center at Stanford (METRICS), Stanford University, Palo Alto, CA 94305 USA; 26 Department of Psychiatry, University of Toronto, Toronto, ON Canada; 27 Centre for Addiction & Mental Health (CAMH), 33 Russel Street, room RS1050S, Toronto, ON M5S 2S1 Canada

**Keywords:** Depression, Mortality, All-cause, Cause-specific, Systematic reviews, Meta-analyses, Survival, Umbrella review, Psychiatry

## Abstract

**Background:**

Depression is a prevalent and disabling mental disorder that frequently co-occurs with a wide range of chronic conditions. Evidence has suggested that depression could be associated with excess all-cause mortality across different settings and populations, although the causality of these associations remains unclear.

**Methods:**

We conducted an umbrella review of systematic reviews and meta-analyses of observational studies. PubMed, PsycINFO, and Embase electronic databases were searched through January 20, 2018. Systematic reviews and meta-analyses that investigated associations of depression and all-cause and cause-specific mortality were selected for the review. The evidence was graded as convincing, highly suggestive, suggestive, or weak based on quantitative criteria that included an assessment of heterogeneity, 95% prediction intervals, small-study effects, and excess significance bias.

**Results:**

A total of 26 references providing 2 systematic reviews and data for 17 meta-analytic estimates met inclusion criteria (19 of them on all-cause mortality); data from 246 unique studies (*N* = 3,825,380) were synthesized. All 17 associations had *P* < 0.05 per random effects summary effects, but none of them met criteria for convincing evidence. Associations of depression and all-cause mortality in patients after acute myocardial infarction, in individuals with heart failure, in cancer patients as well as in samples from mixed settings met criteria for highly suggestive evidence. However, none of the associations remained supported by highly suggestive evidence in sensitivity analyses that considered studies employing structured diagnostic interviews. In addition, associations of depression and all-cause mortality in cancer and post-acute myocardial infarction samples were supported only by suggestive evidence when studies that tried to adjust for potential confounders were considered.

**Conclusions:**

Even though associations between depression and mortality have nominally significant results in all assessed settings and populations, the evidence becomes weaker when focusing on studies that used structured interviews and those that tried to adjust for potential confounders. A causal effect of depression on all-cause and cause-specific mortality remains unproven, and thus interventions targeting depression are not expected to result in lower mortality rates at least based on current evidence from observational studies.

**Electronic supplementary material:**

The online version of this article (10.1186/s12916-018-1101-z) contains supplementary material, which is available to authorized users.

## Background

Major depressive disorder is a chronic and recurring condition with an estimated lifetime prevalence of 14.6% and 11.1% in high- and lower- and middle-income countries, respectively [1, 2]. In addition, major depressive disorder is a leading source of disability worldwide [3, 4], and is associated with diminished quality of life and medical morbidity [2, 4, 5]. An accumulating body of evidence also indicates that major depressive disorder may confer a higher risk for several non-communicable diseases (for example, diabetes [6], obesity [7], stroke [8], acute myocardial infarction [9], dementia [10], and physical health multimorbidity [11]), while these chronic health conditions appear to increase the likelihood of developing depression [7, 12–15].

It has long been suggested that depression is associated with elevated all-cause mortality [16, 17], and is an established risk factor for completed suicide [18]. In addition, depression has been associated with higher mortality rates across several settings and populations, including community samples, inpatients/outpatients, and patients with specific medical conditions (for example, stroke, diabetes, and coronary heart disease) [9, 16, 19, 20]. However, consistent evidence has not shown that specific interventions targeting depression may increase survival in both community and clinical samples. Furthermore, several confounding variables may account for the observed associations between depression and survival, namely sociodemographic variables [21], physical inactivity [22, 23], higher smoking rates [24], follow-up duration of studies [16], and co-occurring medical and psychiatric conditions [5, 25].

Several individual systematic reviews and meta-analyses have investigated the association between depression and mortality across distinct populations (for example, in community samples as well as in samples with specific chronic diseases) [16, 20, 26–28]. To synthesize and evaluate the available evidence we conducted an umbrella review of systematic reviews and meta-analyses that assessed the association of depression and all-cause and cause-specific mortality. The strength of the evidence supporting these associations and hints of bias were evaluated using standardized approaches [8, 29–31].

## Methods

### Literature search

We conducted an umbrella review, which is the systematic collection and assessment of multiple systematic reviews and meta-analyses done in a specific research topic [29]. The PubMed/MEDLINE, EMBASE, and PsycINFO databases were searched from inception up to January 20, 2018, for systematic reviews and meta-analyses of observational studies which examined the association of depression and all-cause or cause-specific mortality. A pre-defined search strategy was used (Additional file 1).

### Eligibility criteria

We included systematic reviews and meta-analyses of observational epidemiological studies performed in humans that assessed the impact of depression on all-cause or cause-specific mortality in any specific population (for example, community samples, samples with a specific medical condition, inpatients, etc.). In addition, systematic reviews and meta-analyses that solely investigated the association of depression and suicide-related deaths were not considered; this was not an aim of the current effort as depression is an established risk factor for completed suicide [18]. However, suicide-related deaths were considered in meta-analyses that estimated the association of depression and all-cause mortality across different populations. No language restrictions were considered for the selection of systematic reviews and meta-analyses for this umbrella review. We included unique observational studies derived from all available systematic reviews and meta-analyses on a specific topic. Whenever a meta-analysis included a lower number of component studies compared to another meta-analysis on the same topic, the former was excluded only if all its individual datasets were included in the larger meta-analysis. Otherwise, we also extracted data from non-overlapping datasets included only in the meta-analysis with fewer studies. This approach aimed to synthesize the largest evidence possible derived from available systematic reviews and meta-analyses. Across each eligible systematic review and/or meta-analysis we considered studies in which the case definition of depression was based on either *International Classification of Disease* [32] (ICD), *Diagnostic and Statistical Manual of Mental Disorders* [33] (DSM), or other consensus-based acceptable criteria (e.g., the Research Diagnostic Criteria [34]). We also included studies where depression was assessed by means of a screening instrument with a specific cutoff score (e.g., the Patient Health Questionnaire-9 and the Beck Depression Inventory). We excluded individual studies from eligible systematic reviews and meta-analyses according to the following criteria: (1) reported an association only for depressive symptoms (i.e., the association was reported for an increase in scores of a depression rating scale instead of a possible diagnosis of depression based on a screening tool with a cutoff point); (2) considered other mental disorders (e.g., dysthymia) in the mortality outcome assessment unless data for depression, as defined above, was provided separately; (3) a diagnosis of depression was based only on clinical evaluation without any specification of the diagnostic criteria; (4) a diagnosis of depression was based only on the use of antidepressants or otherwise on a self-reported (or record-based) history of depression; (5) the association was reported considering other outcomes in addition to mortality (e.g., recurrence); and (6) studies that provided results based on controls that were not included in the original sample (for example, studies that estimated the associations of depression and mortality through standardized mortality ratios compared with general population data external to the study sample).

Two authors (MOM and NV) independently screened the titles and abstracts of retrieved references for eligibility. The full-text articles of potentially eligible articles were then independently scrutinized in detail by two investigators (MOM and NV). Disagreements were resolved through consensus or discussion with a third investigator (CAK or AFC).

### Data extraction

Data extraction was done independently by two investigators (MOM and NV) and, in case of discrepancies, a third investigator made the final decision (CAK and AFC). For each eligible reference, we recorded the first author, year, journal of publication, specific populations evaluated and the number of included studies. If a quantitative synthesis was performed, we also extracted the most fully adjusted study-specific risk estimates (relative risk, odds ratio, hazard ratio, or incident risk ratio) and corresponding 95% confidence intervals (CIs). When available, we also extracted the following variables from each study: number of cases (number of death events in participants with depression), sample size, follow-up time, covariates included in multivariable models, method used to define depression (i.e., structured diagnostic interview or screening instrument), study design (case-control, prospective cohort, or retrospective cohort), specific population, as well as the setting and country where the study was conducted. Whenever studies used several control groups, we considered data from healthy controls as the control group. For studies with no quantitative synthesis, the authors’ main interpretations about their findings and reasons why a meta-analysis was not conducted were recorded.

### Statistical analysis and methodological quality appraisal

We based our analysis on the largest meta-analysis that evaluated the association of depression and all-cause or cause-specific mortality. Furthermore, all datasets from similar meta-analyses that were not included in the largest available one were also considered (i.e., we included all datasets from the smaller meta-analysis that did not overlap with the larger one). We then estimated effect sizes (ES) and 95% CIs through both fixed and random effects models [35]. We also estimated the 95% prediction interval, which further accounts for between-study heterogeneity, and evaluates the uncertainty of the effect that would be expected in a new study addressing the same association [36, 37]. For the largest dataset of each meta-analysis, we calculated the standard error of the ES. If the standard error is < 0.1, then the 95% CI will be < 0.20 (i.e., less than the magnitude of a small ES). We calculated the *I*^*2*^ metric to quantify between-study heterogeneity. Values ≥ 50% indicate large heterogeneity, and values ≥ 75% are indicative of very large heterogeneity [38, 39]. To assess evidence for small-study effects we used the asymmetry test developed by Egger et al. [40]. A *P* value < 0.10 in the Egger’s test and the ES of the largest study being more conservative than the summary random effects ES of the meta-analysis were considered indicative of small-study effects [41]. Finally, evidence of an excess of significance was assessed by the Ioannidis test [42]. Briefly, this test estimates whether the number of studies with nominally significant results (i.e., *P* < 0.05) among those included in a meta-analysis is too large considering their power to detect significant effects at an alpha level of 0.05. First, the power of each study is estimated with a non-central *t* distribution. The sum of all power estimates provides the expected (E) number of datasets with nominal statistical significance. The actual observed (O) number of statistically significant datasets is then compared to the E number using a χ^2^-based test [42]. Since the true ES of a meta-analysis cannot be precisely determined, we considered the ES of the largest dataset as the plausible true ES. This decision was based on the fact that simulations indicate that the most appropriate assumption is the ES of the largest dataset included in the meta-analysis [43]. Excess significance for a single meta-analysis was considered if *P* < 0.10 in Ioannidis’s test and O > E. We graded the credibility of each association with standard approaches on the following categories [31, 44]: convincing (class I), highly suggestive (class II), suggestive (class III), weak evidence, and non-significant associations (Table 1).

**Table 1:** Criteria for classification of the credibility of the evidence (adapted from reference [31])

Classification	Criteria
Convincing evidence (Class I)	More than 1000 death events
	Significant summary associations (*P* < 10^− 6^) per random effects calculations
	No evidence of small-study effects
	No evidence of excess of significance
	Prediction intervals not including the null
	Not large heterogeneity (i.e., *I*^*2*^ < 50%)
Highly-suggestive evidence (Class II)	Significant summary associations (*P* < 10^− 6^) per random effects calculation
	More than 1000 death events
	The largest study with 95% confidence intervals excluding the null
Suggestive evidence (Class III)	More than 1000 death events
	Significant summary associations (*P* < 10^−3^) per random effects calculations
Weak evidence	All other associations with *P* < 0.05
Non-significant associations	All associations with *P* > 0.05

For associations supported by either class I or II evidence, we conducted additional analyses. First, grading of the evidence was re-assessed through sensitivity analyses (when at least three independent datasets were available for each subgroup). The following analyses were considered: (1) prospective cohort studies; (2) studies in which the ascertainment of depression was performed by means of a structured diagnostic interview; (3) studies that provided estimates adjusted for potential confounding variables through multivariable models; (5) studies from which estimates were adjusted at least for sex and age; (6) studies that adjusted for characteristics of the underlying somatic disease (i.e., whenever the association of depression and mortality was assessed in a population with a specific somatic condition); (7) studies that adjusted estimates for the presence of co-morbid diseases (including mental and/or somatic conditions); (8) settings where samples were derived from (community, primary care, outpatient samples, or inpatient samples); and (9) studies in which the follow-up time was longer than 5 years. Finally, we used credibility ceilings, which is a method of sensitivity analyses to account for potential methodological limitations of observational studies that might lead to spurious precision of combined effect estimates. In brief, this method assumes that every observational study has a probability *c* (credibility ceiling) that the true effect size is in a different direction from the one suggested by the point estimate [45, 46]. The pooled effect sizes were re-estimated considering a wide range of credibility ceiling values [30, 45]. All analyses were conducted in STATA/MP 14.0 (StataCorp, USA) with the metan package.

Two investigators (MOM and NV) independently rated the methodological quality of included systematic reviews and meta-analyses with the Assessment of Multiple Systematic Reviews (AMSTAR) instrument, which has been validated for this purpose [47–49]. Scores range from 0 to 11 with higher scores indicating greater quality. The AMSTAR tool involves dichotomous scoring (i.e., 0 or 1) of 11 related items to assess methodological rigor of systematic reviews and meta-analyses (e.g., comprehensive search strategy, publication bias assessment). AMSTAR scores are graded as high (8–11), medium (4–7), and low quality (0–3) [47].

## Results

**Fig. 1:**
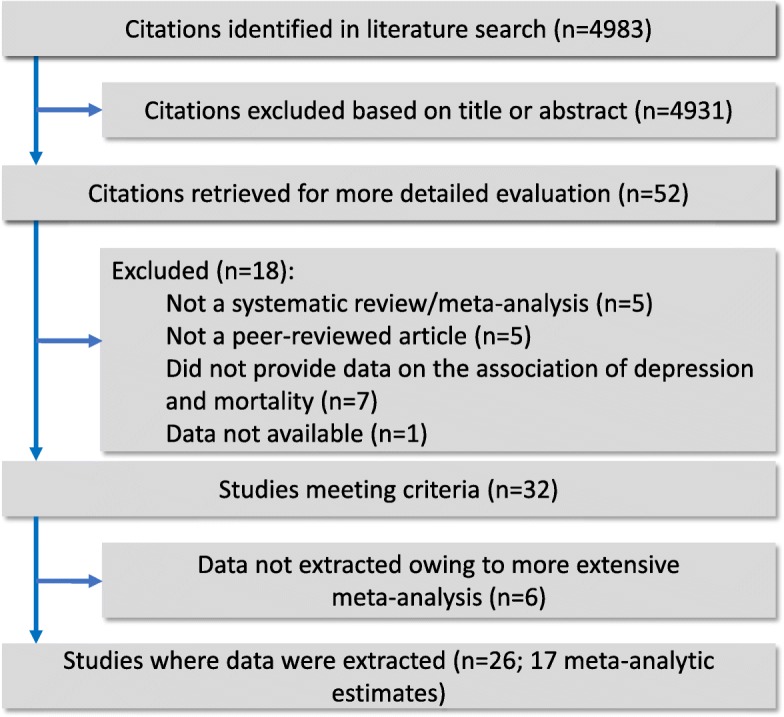
Study flowchart

Overall, the title and abstract of 4983 references were screened for eligibility. The full-text of 52 references were then scrutinized in detail, of which 19 were excluded with reasons (Additional file 1: Table S1), while 26 references met inclusion criteria (Fig. 1). Overall, 24 references provided quantitative synthesis of evidence [16, 19, 20, 26–28, 50–67], and 2 references were qualitative systematic reviews [68, 69]. This umbrella review included 238 prospective studies and 8 retrospective cohort studies and comprised data from 3,825,380 participants, including 293,073 participants with depression and 282,732 death events, which were grouped in 17 meta-analytic estimates (Additional file 1: Table S2). Overall, 246 eligible studies were derived from included meta-analyses, while 667 component studies were excluded from eligible meta-analyses due to the following reasons: datasets were included in more than one meta-analysis (*k* = 375); other mental disorders (e.g., dysthymia) were considered in the association between depression and mortality (*k* = 14); a diagnosis of depression was based only on clinical evaluation without any specification of the diagnostic criteria (*k* = 7); a diagnosis of depression was based only on the use of antidepressants (*k* = 5); the association included other outcomes besides mortality (e.g., recurrence) (*k* = 5); overlapping samples (*k* = 20); did not provide data for ES estimation (*k* = 12); a diagnosis of depression was not established according to inclusion criteria (*k* = 223); and assessed the impact of depression on mortality considering standardized mortality ratios against general population data external to the study (*k* = 6). Overall, 165 studies (67.1%) provided adjusted association metrics, with a median number of 5 (IQR 3–8) covariates controlled for in multivariable models (see Additional file 1: Table S3 for the list of factors that were considered in multivariable models in studies derived from eligible meta-analyses). The median follow-up time of included studies was 4.5 years (IQR 2–7.5). The median AMSTAR score of eligible systematic reviews and meta-analyses was 6 (IQR 5–7.5). Scores of each domain of the AMSTAR instrument are provided in Additional file 1: Table S4.

### Evidence from qualitative systematic reviews

A systematic review that included 3 studies suggested that depression could be associated with reduced long-term survival in patients with head and neck cancers [68]. In addition, a systematic review that included 11 studies that assessed the association of depression and mortality in chronic pulmonary obstructive pulmonary disease (COPD) met inclusion criteria. The authors concluded that depression could be associated with an increase in early mortality in patients with COPD [69].

### Summary effect sizes

At a threshold of *P* < 0.05, summary ESs were significant for all 17 (100%) meta-analytic estimates in both fixed and random effects models (Additional file 1: Table S2). At a more conservative threshold of *P* < 0.001, 16 (94.1%) and 9 (52.9%) estimates were significant in fixed and random effects models, respectively. At a threshold of *P* < 10^− 6^, 12 (70.6%) and 5 (29.4%) meta-analyses were statistically significant in fixed and random effects models, respectively.

### Heterogeneity between studies

Six meta-analyses (35.6%) showed large heterogeneity (I^2^ = 50–75%) and 5 (29.4%) exhibited very large heterogeneity (I^2^ > 75%) (Additional file 1: Table S5). We further assessed the uncertainty of the summary effects by calculating their 95% prediction intervals; the null value was excluded in only 3 associations, namely in all-cause mortality in coronary artery bypass graft patients, coronary heart disease patients, and COPD patients.

### Small-study effects

Evidence of small-study effects was verified in 13 meta-analyses, including associations of depression and all-cause mortality in patients after coronary artery bypass grafting, with acute coronary syndrome or coronary heart disease, after stroke, post-transplant patients, and people with HIV, chronic kidney disease, heart failure, COPD, diabetes mellitus, and mixed settings, as well as associations with depression and fatal stroke and cardiovascular mortality after acute myocardial infarction (Additional file 1: Table S5) [51].

### Excess significance

We assessed excess of significance bias (i.e., the likelihood that the observed number of nominally significant studies could exceed the expected number of ‘positive’ studies for a given estimate). Eleven (64.7%) meta-analyses had evidence of excess significance bias, namely those investigating the associations of all-cause mortality and cancer, heart failure, mixed settings, coronary heart disease, acute coronary syndrome, stroke, post-transplant patients, chronic kidney disease, as well as associations of depression and fatal stroke, cardiovascular mortality in patients with diabetes mellitus, and cardiovascular mortality in mixed settings (Additional file 1: Table S5).

### Grading of the evidence

We explored whether the nominally significant associations between mortality and depression were supported by convincing, highly suggestive, suggestive, or weak evidence (Table 2). Overall, no association was supported by convincing evidence, while associations of depression and all-cause mortality among patients with cancer, patients after acute myocardial infarction, patients with heart failure, and mixed settings (including inpatients, outpatients, and community as well as primary care samples) were supported by highly suggestive evidence. Furthermore, associations between depression and all-cause mortality in patients with coronary heart disease and diabetes mellitus were supported by suggestive evidence. Finally, the remaining 11 (64.7%) associations were supported by weak evidence (Table 2).

**Table 2:** Details of evidence grading for meta-analyses investigating associations of depression and mortality

Author, year	Population	Mortality type	Sample size MDD/Deaths	k	Largest study ES (95% CI)^a^	Random effects summary RR^b^ (95% CI)	Random effects *P* value^c^	95% prediction interval	I^2^ (%)	Excess significance O/E^d^	Excess significance *P*- value
Associations supported by highly suggestive evidence											
Cuijpers, 2014 [51] Satin, 2009 [62]	Cancer	All-cause	4034/4817	23	1.37 (1.26–1.50)	1.55 (1.32–1.81)	< 10^−6^	0.80–2.50	69.6	11/4.53	< 0.001
Cuijpers, 2014 [51] Sorensen, 2005 [64] Meijer, 2013 [67] van Melle, 2004 [58]	Post-AMI	All-cause	4183/2358	20	1.48 (1.12–1.96)	2.09 (1.66–2.63)	< 10^−6^	0.89–3.54	66.6	12/11.0	0.64
Cuijpers, 2014 [51] Fan, 2014 [28] Gathright 2017 [54] Sokoreli, 2016 [63]	HF	All-cause	3418/4345	22	1.33 (1.19–1.42)	1.46 (1.30–1.65)	< 10^−6^	0.89–1.92	81.0	14/6.5	< 0.001
Baxter, 2011 [50] Cuijpers, 2002 [19] Cuijpers, 2014 [51] Walker, 2015 [16]	Mixed-sample^e^	All-cause	87,633/242577	111	1.77 (1.41–2.17)	1.48 (1.39–1.58)	< 10^−6^	0.84–2.23	89.3	55/17.8	0.00
Associations supported by suggestive evidence											
Barth, 2004 [26] Cuijpers, 2014 [51] Leung, 2012 [56] Meijer, 2013 [67]	CHD	All-cause	2284/1533	10	1.21 (1.04–1.42)	1.57 (1.27–1.94)	< 10^−4^	1.16–1.47	62.6	5/1.18	< 0.001
Cuijpers, 2014 [51] Hofmann, 2013 [55] Park, 2013 [60]	DM	All-cause	4373/7452	12	1.06 (0.96–1.18)	1.60 (1.30–1.80)	< 10^−6^	0.90–3.21	83.6	8/5.72	0.18
Associations supported by weak evidence											
Cuijpers, 2014 [51] Stenman, 2016 [65]	CABG	All-cause	503/347	4	2.40 (1.40–4.00)	1.93 (1.43–2.60)	< 10^−4^	1.09–3.18	0	2/2.32	0.74
Cuijpers, 2014 [51] Meijer, 2013 [67]	ACS	All-cause	324/163	3	2.80 (1.40–5.07)	1.82 (1.02–3.26)	0.04	0.06–48.2	86.4	2/0.54	0.02
Bartoli, 2013 [20] Cuijpers, 2014 [51] Pan, 2011 [59]	Stroke	All-cause	3103/414	7	1.13 (1.06–1.21)	1.46 (1.15–1.85)	0.002	0.80–2.13	62.1	4/0.04	0.00
Pan, 2011 [59]	Stroke	Fatal Stroke	1600/377	4	1.66 (1.16–2.39)	1.58 (1.00–2.50)	0.049	0.21–8.07	46.7	2/0.02	0.00
Cuijpers, 2014 [51] Dew, 2015 [52]	Post-transplant patients^f^	All-cause	405/433	6	1.66 (1.12–2.47)	1.64 (1.37–1.95)	< 10^−2^	0.71–2.65	36.5	4/0.86	< 10^− 3^
Cuijpers, 2014 [51]	HIV	All-cause	1977/1580	4	1.60 (1.32–1.92)	1.30 (1.05–1.61)	0.017	0.61–2.70	55.1	1/1.79	0.42
Meijer, 2011 [57]	AMI	Cardiovascular Mortality	995/114	5	5.51 (0.61–49.18)	2.98 (1.65–5.38)	< 10^−3^	0.26–15.80	42.3	3/2.37	0.57
Cuijpers, 2014 [51] Palmer, 2013 [27]	CKD	All-cause	922/930	12	0.98 (0.72–1.34)	1.66 (1.20–2.30)	< 10^−2^	0.84–1.40	44.4	4/0.17	0.00
Cuijpers, 2014 [51]	COPD	All-cause	338/261	5	1.93 (1.04–3.58)	2.34 (1.69–3.24)	< 10^−6^	1.23–3.63	0.00	4/3.01	0.25
van Dooren, 2013 [53]	DM	Cardiovascular	1255/536	4	1.25 (0.83–1.86)	1.48 (1.08–2.03)	0.014	0.61–3.00	52.5	2/0.38	< 10^−2^
Correll, 2017 [66]	Mixed samples^g^	Cardiovascular mortality	175,726/14495	4	1.00 (0.85–1.17)	1.56 (1.08–2.24)	0.018	0.34–6.82	87.8	3/1.00	0.02

*ACS* acute coronary syndrome, *AMI* acute myocardial infarction, *CA* cancer, *CABG* coronary artery bypass grafting, *CHD* coronary heart disease, *CI* confidence interval, *CKD* chronic kidney disease, *COPD* chronic pulmonary obstructive disease, *DM* diabetes mellitus, *E* expected, *ES* effect size, *HF* heart failure, *MDD* major depressive disorder, *O* observed, *RR* risk ratio

^a^Relative risk and 95% confidence interval of largest study (smallest standard error) in each meta-analysis

^b^Random effects refer to summary effect size (95% confidence interval) using the random effects model

^c^*P* value of summary random effects estimate

^d^Expected number of statistically significant studies using the point estimate of the largest study (smallest standard error) as the plausible effect size

^e^Kidney, liver, heart, and lung transplantation

^f^Includes community samples, inpatients, outpatients, and primary care samples

^g^Includes community, outpatient, and inpatient samples

### Sensitivity analyses

Sensitivity analyses were performed for the four associations supported by highly suggestive evidence as per our protocol (Table 3). It is worth noting that, when studies that employed structured/semi-structured diagnostic interviews were considered, associations of depression and all cause-mortality in cancer as well as post-acute myocardial infarction became supported by weak evidence, while the association of depression and all-cause mortality in mixed settings dropped to suggestive evidence. Furthermore, when only studies that provided adjusted estimates were considered, associations of depression and all-cause mortality in cancer and post-acute myocardial infarction dropped to suggestive evidence. Moreover, the association of depression and all-cause mortality in cancer was supported by suggestive evidence only when studies that adjusted at least for age and sex were assessed in analysis.

**Table 3:** Sensitivity analyses for associations of depression and all-cause mortality supported by highly suggestive (class II) evidence

Subgroup	Sample size MDD/Deaths	k	Largest study ES (95% CI)^a^	Random effects summary ES^b^ (95% CI)	Random effects *P* value^c^	95% prediction interval	I^2^ (%)	Excess significance		Classification
								O/E^d^	*P* value	
All-cause in cancer										
Structured interview	145/462	5	2.85 (2.29–3.54)	1.56 (0.87–2.8)	0.133	0.28–8.56	71.9	1/1.02	0.979	Weak
Adjusted estimates only	1066/2273	13	1.2 (0.9–1.4)	1.6 (1.35–1.9)	< 10^−6^	1.02–2.51	42.3	8/5.16	0.097	Class III
Adjusted at least for age and sex	691/1030	6	1.2 (0.9–1.4)	1.69 (1.23–2.31)	0.001	0.77–3.68	51.6	4/3.3	0.563	Class III
Adjusted comorbidities	910/1951	9	1.2 (0.9–1.4)	1.52 (1.27–1.83)	< 0.001	0.97–2.4	43.3	5/3.54	0.3	Class III
Inpatients	764/618	10	1.66 (1.16–2.37)	1.7 (1.35–2.13)	< 0.001	0.96–3	40.9	6/3.55	0.106	Weak
Outpatients	453/1418	10	1.3 (0.98–1.73)	1.56 (1.11–2.17)	0.009	0.57–4.28	75.3	4/2.57	0.291	Class III
Prospective studies	4034/4817	23	1.37 (1.26–1.5)	1.55 (1.32–1.81)	< 10^−6^	0.86–2.78	69.6	11/6.21	0.023	Class II
Follow-up ≤ 5 years	946/1580	15	1.2 (0.9–1.4)	1.8 (1.42–2.28)	< 0.001	0.83–3.9	68.1	9/6.64	0.22	Class III
Follow-up > 5 years	3088/3237	8	1.37 (1.26–1.5)	1.29 (1.14–1.47)	< 0.001	1–1.68	22.5	2/1.52	0.658	Class III
All-cause in heart failure patients^e^										
Adjusted estimates only	3383/4275	21	1.33 (1.19–1.42)	1.46 (1.29–1.64)	< 10^−6^	0.93–2.27	79.1	14/9.83	0.068	Class II
Adjusted at least for age and sex	2526/2935	13	1.33 (1.19–1.42)	1.36 (1.22–1.52)	< 10^−6^	0.99–1.86	54.3	9/5.39	0.042	Class II
Adjusted for comorbidities	2395/3371	12	1.33 (1.19–1.42)	1.43 (1.26–1.62)	< 10^−6^	1–2.04	60.5	8/6.58	0.41	Class II
Inpatients	1245/1500	7	2.02 (1.48–2.76)	1.82 (1.28–2.6)	< 0.001	0.64–5.19	77.7	4/5.12	0.339	Class III
Outpatients	639/583	6	1.31 (1.07–1.6)	1.46 (1.08–1.96)	0.013	0.67–3.16	75.2	4/1.8	0.049	Weak
Prospective studies	3418/4345	22	1.33 (1.19–1.42)	1.46 (1.3–1.65)	< 10^−6^	0.94–2.28	78.4	14/10.02	0.088	Class II
Follow-up ≤ 5 years	2417/2358	16	1.33 (1.19–1.42)	1.52 (1.3–1.77)	< 10^−6^	0.93–2.47	80.0	10/6.55	0.079	Class II
Follow-up > 5 years	1001/1987	6	1.31 (1.07–1.6)	1.4 (1.14–1.72)	0.001	0.76–2.57	72.5	4/3.41	0.627	Class III
All-cause in mixed sample^e^										
Structured interview	4746/29667	19	2.3 (2.1–2.5)	1.64 (1.3–2.08)	< 0.001	0.62–4.38	88.7	11/13.06	0.277	Class III
Adjusted estimates only	83,470/212385	81	1.77 (1.41–2.17)	1.42 (1.33–1.5)	< 10^−6^	0.93–2.15	86.5	44/16.69	0	Class II
Adjusted at least for age and sex	51,332/161660	42	1.1 (1.07–1.13)	1.34 (1.25–1.43)	< 10^−6^	0.96–1.85	82.4	23/15.72	0.017	Class II
Adjusted for comorbidities	34,122/41488	53	1.77 (1.41–2.17)	1.38 (1.29–1.47)	< 10^−6^	0.97–1.96	71.6	31/3.66	0	Class II
Community	32,269/69181	62	1.77 (1.41–2.17)	1.48 (1.36–1.61)	< 10^−6^	0.83–2.63	88.2	31/8.76	0	Class II
Inpatients	2209/2334	16	1.44 (1.1–1.88)	1.58 (1.33–1.87)	< 10^−6^	0.93–2.68	56.0	10/5.69	0.019	Class II
Outpatients	811/497	6	1.55 (1.06–2.26)	1.47 (1.13–1.91)	0.004	0.8–2.68	34.5	4/0.58	0	Weak
Primary care	8730/4558	6	1.04 (0.93–1.15)	1.44 (1.11–1.86)	0.006	0.67–3.1	82.0	4/2.13	0.11	Class III
Prospective studies	46,951/96860	95	1.77 (1.41–2.17)	1.51 (1.4–1.62)	< 10^−6^	0.86–2.63	87.1	51/11.97	0	Class II
Follow-up ≤ 5 years	24,944/26135	61	1.37 (1.19–1.48)	1.62 (1.48–1.77)	< 10^−6^	0.96–2.71	75.7	34/17.79	0	Class II
Follow-up > 5 years	62,689/216442	40	1.77 (1.41–2.17)	1.36 (1.24–1.48)	< 10^−6^	0.81–2.26	93.8	21/8.41	0	Class II
All-cause in post-AMI										
Structured interview	1688/638	5	1.48 (1.12–1.96)	2.37 (1.36–4.14)	0.002	0.41–13.76	86.3	4/4.12	0.886	Weak
Adjusted estimates only	2381/1771	9	1.48 (1.12–1.96)	2.2 (1.51–3.2)	< 0.001	0.71–6.81	80.7	7/7.88	0.374	Class III
Adjusted for comorbidities	1507/579	3	1.48 (1.12–1.96)	1.56 (1.18–2.06)	0.001	0.79–3.1	5.1	2/2.15	0.843	Weak
Inpatients	3998/2196	17	1.48 (1.12–1.96)	2.09 (1.63–2.69)	< 10^−6^	0.9–4.85	70.3	10/12.89	0.102	Class II
Prospective studies	4183/2358	20	1.48 (1.12–1.96)	2.09 (1.66–2.63)	< 10^−6^	0.95–4.62	66.6	12/14.72	0.168	Class II
Follow-up ≤ 5 years	3602/1789	16	1.67 (1.31–2.12)	2.18 (1.66–2.86)	< 10^−6^	0.89–5.32	69.4	9/12.4	0.042	Class II
Follow-up > 5 years	533/560	3	1.48 (1.12–1.96)	1.57 (1.25–1.99)	< 0.001	0.94–2.63	0.0	2/1.31	0.42	Weak

*AMI* acute myocardial infarction, *CI* confidence interval, *E* expected, *ES* effect size, *MDD* major depressive disorder, *NA* not available, *NE* not evaluated, *NS* not significant, *O* observed

^a^ES and 95% confidence interval of largest study (smallest standard error) in each meta-analysis

^b^Random effects refer to summary effect size (95% CI) using the random effects model

^c^P value of summary random effects estimate

^d^Expected number of statistically significant studies using the point estimate of the largest study (smallest standard error) as the plausible effect size

^e^Include community samples, inpatients, outpatients and primary care

Sensitivity analyses through credibility ceilings were also conducted for the four associations supported by highly suggestive evidence (Additional file 1: Table S6). All associations remained significant when 10% credibility ceilings were considered, while no associations were nominally significant when 20% credibility ceilings were considered.

## Discussion

The associations between mental disorders and mortality have been investigated for more than 150 years [70, 71]. The associations between depression and all-cause and cause-specific mortality has been particularly investigated across different types of settings and populations. All meta-analyses have obtained nominally statistically significant results for a higher risk of mortality in almost all the tested populations. However, no associations met criteria for convincing evidence, while only four associations, namely those of depression and all-cause mortality in cancer, heart failure, mixed settings as well as among patients after acute myocardial infarction, were supported by highly suggestive evidence. Nevertheless, our sensitivity analyses indicate that differences in case ascertainment of depression as well as the lack of proper adjustment for confounding variables and other major risk factors could render several associations supported by lower levels of evidence. Therefore, the current work suggests that causal inferences between depression and all-cause mortality across distinct populations do not appear to be as conclusive as once thought [16, 21, 72].

Several variables and mechanisms may contribute to the observed associations of depression and all-cause mortality. Some effects may be direct. For example, it has been suggested that depression activates several pathophysiological mechanisms that could contribute to the emergence of chronic somatic diseases that are consistently related to lowered survival. For instance, it has been claimed that depression is associated with peripheral inflammation [73] and oxidative stress [74], mechanisms which may contribute to the association of depression and obesity and cardio-metabolic conditions [66, 75–77]. However, depression may also exert indirect effects on survival. For example, a large body of evidence suggests that depression alters illness behavior [78], leading to a meaningful decrease in treatment adherence across several conditions [79, 80] as well as unhealthy lifestyles (e.g., sedentary behavior, higher prevalence of smoking, and non-salutary diet) [23, 73, 81, 82]. Depression also often co-exists with other mental health conditions that may also be associated with elevated mortality rates [25, 72]. Multivariable adjustment has varied across included studies, and only approximately 40% of included studies controlled their results at least for age and sex. Mortality analyses that do not account for at least these two major determinants of death risk are problematic. We observed that, when only studies that controlled for age and sex were considered, the association of depression and all-cause mortality in cancer was no longer supported by highly suggestive evidence. Furthermore, no association was supported by highly suggestive evidence when only studies that employed structured/semi-structured diagnostic interviews were considered. This is a relevant finding since recent evidence suggests that the selective use of different cutoff points may bias accuracy estimates of screening instruments for depression, even if these instruments are considered to be validated, whilst this type of bias does not apparently occur in gold-standard structured diagnostic interviews [83]. It is worthy to note, however, that the association between depression and all-cause mortality among patients with heart failure remained supported by highly suggestive evidence when only studies that provided either adjusted estimates or, otherwise, that adjusted for age and sex were considered, while due to the lack of available datasets sensitivity analyses considering studies that used structured/semi-structured diagnostic interviews could not be performed. Therefore, further studies should be conducted to evaluate this association.

### Comparison with other studies

Cuijpers et al. [51] performed the largest meta-analysis to date assessing the impact of depression on mortality. Although this previous meta-analysis concluded that depression is associated with all-cause mortality, fewer studies were available when that study was conducted. In addition, the inclusion criteria differed from ours. For example, Cuijpers et al. [51] included studies in which a diagnosis of depression was based on previous exposure to antidepressants, which are drugs used for several other medical and psychiatric indications, whilst we limited our inclusion criteria to investigations in which depression was assessed by either a structured/unstructured diagnostic interview or a screening instrument with a cut-off score, and also large-scale studies that used a coded diagnosis of depression based on well-established criteria. In addition, we estimated the credibility of the evidence in different settings and populations with state-of-the art statistical methods used in previous umbrella reviews [8, 30].

A previous meta-review investigated the associations between severe mental disorders (including depression) and all-cause and suicide-related mortality [72]. Although the authors concluded that depression was associated with an excess of all-cause mortality, only three references were included and the credibility of the evidence was not quantitatively assessed. Finally, a recent study pooled evidence from 15 systematic reviews and meta-analyses and observed that evidence that depression is associated with all-cause mortality remains inconclusive [84]. This previous effort is the most comprehensive assessment of the impact of depression on mortality conducted to date. The inclusion criteria differed from ours. Furthermore, in the current effort, an attempt to demarcate the putative impact of depression on survival in different populations was performed. In addition, we assessed several hints of biases in this literature. Our findings provide further quantitative evidence that the causality of associations between depression and elevated all-cause mortality across different populations and settings remains to be proven.

### Strengths and limitations

Our umbrella review might have missed some available evidence, e.g., recently published studies that had not been included in the prior meta-analyses [29]. However, in this effort, we assessed all available systematic reviews and meta-analyses, and all unique datasets which met inclusion criteria were synthesized for each estimate from all available meta-analyses and most considered meta-analyses were very recent. Although several hints of bias were found to be prevalent in this literature, it is relevant to mention that this finding does not exclude the presence of genuine (i.e., true) heterogeneity in this field. Moreover, the Ioannidis test has relatively low power in a context of high heterogeneity [42], while the assumption that the largest study could approximate the underlying ‘true’ effect size of a meta-analysis may be less straightforward for observational studies than for randomized controlled trials. Depression is a heterogeneous phenotype with different symptomatic dimensions and subtypes [85]. For example, a model has proposed that the duration and specific dimensions of depression (i.e., ‘cognitive/affective’ versus ‘somatic/affective’) may have a differential impact on the progression of coronary artery disease after acute coronary syndrome [86]. This framework was supported by a previous meta-analysis that has shown that somatic/affective symptoms of depression may exert a stronger deleterious effect upon mortality compared to cognitive/affective symptoms in patients with heart disease [87]. In addition, a recent individual-patient meta-analysis suggested that, following proper adjustment for cardiovascular factors, the association between depression and all-cause mortality is notably attenuated in patients after an acute myocardial infarction [67]. This finding underscores that the extent of proper or suboptimal adjustment of clinical and sociodemographic variables may render the association between depression and mortality less consistent across populations with chronic diseases. Although we conducted several sensitivity analyses, the reporting and multivariable adjustment to potential confounders was not consistent across included studies, thus limiting the quality of available evidence. It is possible that more studies adjusted their results at least to age and sex but considered it so trivial that they did not even report on this. Therefore, more thorough reporting of model specification and adjustment is needed in future studies.

Finally, depression may manifest in samples with chronic somatic conditions differently. For example, the diagnosis of depression in cancer patients has been a matter of debate, and may also be ascribed as a spectrum of syndromes [88, 89], some of which may not be properly captured by conventional diagnostic criteria (e.g., DSM-5 or ICD-10) [88]. Furthermore, there is a spectrum related to the timing of appearance with symptoms. In some circumstances, depression may either antedate or be considered an initial manifestation of chronic somatic diseases [78, 90], whilst in other circumstances depression may occur after the onset of the medical condition [78], and also as a result of treatment and its complications. The current effort could not elucidate how the temporal relationship between depression and the respective chronic medical condition could potentially influence mortality rates.

### Implications

Our findings suggest that available evidence does not consistently allow the establishment of causal inferences linking depression to all-cause and cause-specific mortality across different settings and populations. Yet, the association of depression and all-cause mortality appears to be complex, and may be influenced by several sociodemographic and clinical variables. Moreover, we do not question the association between depression and suicide where the evidence is unquestionable [18, 91, 92]. However, suicides appear to account for a relatively smaller fraction of deaths compared to natural causes of death among people with depression [93–95].

The current data may also reconcile some controversies in existing literature. For example, although previous evidence has suggested that post-acute myocardial infarction depression might be associated with diminished survival, no conclusive evidence indicated that the treatment of depression translates to an increased survival in this specific population [96, 97]. Therefore, findings from this umbrella review of observational studies and data from intervention studies conducted to date appear to concur in that associations between depression and all-cause and cause-specific mortality are unlikely to be causal.

For other conditions, such as cancer, it remains unclear if prevention and treatment of depression may increase overall survival. Management of depression is worthwhile for various other reasons, e.g., improvement of quality of life, but not with the expectation that death risk will decrease. Furthermore, interventions aiming to promote a healthy lifestyle as well as the proper care of co-occurring somatic conditions in those with depression may also lead to a decrease in all-cause mortality [25]. However, the impact of those interventions at an individual, societal, and health system levels upon all-cause survival warrant further investigation.

## Conclusions

The associations between depression and all-cause and specific natural cause mortality has been extensively investigated in a wide range of populations and settings. However, this umbrella review of observational studies indicates that the evidence for causal associations of depression and all-cause mortality remains inconclusive. To draw firmer conclusions, further prospective and collaborative studies with transparent a priori-defined protocols and a proper multivariable adjustment to confounders and other important risk determinants for mortality are warranted.

## Additional file

Additional file 1: Supplementary online text and tables. Search string used; studies excluded, with reasons (**Table S1**); description of 23 meta-analytic estimates of the associations of depression and mortality across different populations (**Table S2**); adjustment of individual studies (**Table S3**); AMSTAR quality assessment (**Table S4**); evaluation of heterogeneity, small-study effects, and excess significance bias (**Table S5**); sensitivity analyses using credibility ceilings (**Table S6**). (DOCX 250 kb)

## Abbreviations

AMSTAR: Assessment of Multiple Systematic Reviews
CI: confidence interval
COPD: chronic pulmonary obstructive disease
E: expected
ES: effect size
O: observed
