# Supplementary material for: The association of depression and all-cause and cause-specific mortality: an umbrella review of systematic reviews and meta-analyses
Source: BMC Med. 2018 Jul 20;16:112. doi: 10.1186/s12916-018-1101-z (PMC6053830; doi:10.1186/s12916-018-1101-z)
Supplement: Supplementary file 1 — Supplementary online text and tables. Search string used; studies excluded, with reasons (Table S1); description of 23 meta-analytic estimates of the associations of depression and mortality across different populations (Table S2); adjustment of individual studies (Table S3); AMSTAR quality assessment (Table S4); evaluation of heterogeneity, small-study effects, and excess significance bias (Table S5); sensitivity analyses using credibility ceilings (Table S6). (DOCX 250 kb) [file 12916_2018_1101_MOESM1_ESM.docx]

**SEARCH STRINGS (from inception up to January 20^th^, 2018)**

**MEDLINE/Pubmed**

((((("Depression"[Mesh] OR "Depressive Disorder"[Mesh])) OR (depression[Title/Abstract] OR unipolar depression[Title/Abstract] OR depress*[Title/Abstract] OR major depressive disorder[Title/Abstract] OR major depression[Title/Abstract] OR unipolar depression[Title/Abstract]))) AND (((death[tiab] OR survival[tiab] OR deaths[tiab] OR mortality[tiab])) OR ((((("Mortality"[Mesh]) OR "Death"[Mesh]) OR "Survival"[Mesh]) OR "Survival Analysis"[Majr]) OR "Prognosis"[Mesh]))) AND systematic[sb]

**EMBASE**

Search 1: depression.sh.

Search 2: depressive disorder.sh.

Search 3: (depression or unipolar depression or major depressive disorder or major depression).ti,ab,kw.

Search 4: #1 or #2 or #3

Search5: mortality.sh.

Search6: death.sh.

Search7: survival.sh.

Search8: (mortality or excess mortality or survival or death$).ti,ab,kw.

Search9: #5 or #6 or #7 or #8

Search10: systematic review.pt,sh.

Search11: meta-analysis.pt,sh.

Search12: (systematic review or meta-analysis or meta-analytic review or meta-analytic).ti,ab,kw.

Search13: #10 or #11

Search 14: #4 and #9 and #13

**PsycINFO**

Search 1: depression.sh.

Search 2: depressive disorder.sh.

Search 3: (depression or unipolar depression or major depressive disorder or major depression).ti,ab,kw.

Search 4: #1 or #2 or #3

Search5: mortality.sh.

Search6: death.sh.

Search7: survival.sh.

Search8: (mortality or excess mortality or survival or death$).ti,ab,kw.

Search9: #5 or #6 or #7 or #8

Search10: systematic review.pt,sh.

Search11: meta-analysis.pt,sh.

Search12: (systematic review or meta-analysis or meta-analytic review or meta-analytic).ti,ab,kw.

Search13: #10 or #11

Search 14: #4 and #9 and #13

**Table S1. Reasons for exclusion of each study.**

| **Reference** | **Reason for exclusion** |
| --- | --- |
| Ayerbe, 2013 [1] | Overlapping meta-analysis |
| Ayerbe, 2013 [2] | Overlapping meta-analysis |
| Bartoli, 2013 [3] | Conference abstract |
| Bartoli, 2014 [4] | Editorial |
| Charlson, 2013 [5] | Did not provide data on the association of depression and mortality |
| Coutwright [6] | Did not provide data on the association of depression and mortality; only depressive symptoms |
| Cole, 2007 [7] | Overlapping meta-analysis |
| Doyle, 2015 [8] | Did not provide data on the association of depression and mortality |
| Engelmann, 2016 [9] | Did not provide data on the association of depression and mortality |
| Ghoneim, 2016 [10] | Not a meta-analysis |
| Lichtman, 2014 [11] | Not a meta-analysis |
| Malberg, 2009 [12] | Letter |
| Marzouka, 2010 [13] | Conference abstract |
| Mitchell, 2016 [14] | Conference abstract |
| Park, 2012 [15] | Not a meta-analysis |
| Pinquart, 2010 [16] | Data not available |
| Shi, 2016 [17] | Conference abstract |
| Van den Akker, 2003 [18] | Overlapping meta-analysis |
| Wu, 2016 [19] | Overlapping meta-analysis |
| Wyman, 2013 [20] | Dissertation/Thesis Abstract |
| Maslej, 2017 [21] | Did not provide data on the association of mortality and depression |
| Liu, 2017 [22] | Did not provide data on the association of mortality and depression |
| Aitken, 2017 [23] | Did not provide data on the association of mortality and depression |
| Shi, 2016 [17] | Overlapping meta-analysis |

**Abbreviation: MA,** meta-analysis

| **Table S2.** **Description of 16 meta-analytic estimates of the associations of depression and mortality across different populations.** | | | | | | | | | | | | |
| --- | --- | --- | --- | --- | --- | --- | --- | --- | --- | --- | --- | --- |
| **Author, year** | **Study Population** | **Mortality Type** | **k** | **Follow-up^α^** **(median, lower, higher)** | **Definition of Depression** | **Sample size (MDD/**  **Deaths)** | **RR Fixed Effects**^†^ | **RR Random effects**^§^ | **RR Largest Study** | **Fixed**  **P-value**^¶^ | **Random P-value**^†^ | **95% Prediction interval** |
| Cuijpers, 2014 [24] Hofmann, 2013 [25] Park, 2013 [26] | DM | All-cause mortality | 12 | 7.6 (10–3) | CBCD, VSI, | 4373/7452 | 1.45  (1.35–1.55) | 1.51  (1.25–1.82) | 1.6  (1.3–1.8) | < 10^-6^ | < 0.001 | 0.69–3.21 |
| Van Dooren, 2013 [27] | DM | Cardiovascular mortality | 4 | 7  (8–5) | VSI | 1255/536 | 1.46  (1.18–1.8) | 1.48  (1.08–2.03) | 1.25  (0.83–1.86) | < 0.001 | 0.014 | 0.61–3 |
| Cuijpers, 2014 [24]  Sorensen, 2005 [28]  van Melle, 2004 [29]  Meijer, 2013 [30] | Post AMI | All-cause mortality | 20 | 2  (10–0.3) | VSI, SI | 4183/2358 | 1.98  (1.79–2.21) | 2.09  (1.66–2.63) | 1.48  (1.12–1.96) | < 10^-6^ | < 10^-6^ | 0.9–3.54 |
| Meijer, 2011 [31] | Post AMI | Cardiovascular mortality | 5 | 1  (10–0.5) | VSI, | 995/114 | 2.87 (1.88–4.4) | 2.98  (1.65–5.38) | 5.51 (0.61–49.18) | < 0.001 | < 0.001 | 0.26–15.81 |
| Bartoli, 2013 [32] Cuijpers, 2014 [24 ] Pan, 2011 [33] | Stroke | All-cause mortality | 7 | 3  (12–1.25) | CBCD, VSI, SI | 3103/414 | 1.17  (1.1–1.25) | 1.46  (1.15–1.85) | 1.13  (1.06–1.21) | < 10^-6^ | 0.002 | 0.81–2.14 |
| Pan, 2011 [33] | Stroke | Fatal Stroke | 4 | 13  (29–7.5) | VSI, SI | 1600/377 | 1.61  (1.22–2.13) | 1.58  (1–2.5) | 1.66  (1.16–2.39) | < 0.001 | 0.05 | 0.21–8.08 |
| Cuijpers, 2014 [24]  Meijer, 2013 [30] | ACS | All-cause mortality | 3 | 1.5  (8–1) | CBCD, VSI, SI | 324/163 | 1.21  (1.12–1.31) | 1.82  (1.02–3.26) | 2.8  (1.4–5.7) | < 0.001 | 0.04 | 0.07–48.21 |
| Barth, 2004 [34]  Cuijpers, 2014 [24]  Leung, 2012 [35]  Meijer, 2013 [30] | CHD | All-cause mortality | 10 | 3.3  (15.2–1) | SI, VSI | 2284/1533 | 1.38  (1.25–1.52) | 1.57  (1.27–1.94) | 1.21 (1.04–1.42) | < 10^-6^ | < 0.001 | 1.16–1.47 |
| Cuijpers, 2014 [24]  Stenman, 2016 [36] | CABG | All-cause mortality | 4 | 5.5  (11.5–5) | VSI | 503/347 | 1.93  (1.43–2.6) | 1.93  (1.43–2.6) | 2.4  (1.4–4) | < 0.001 | < 0.001 | 1.09–3.18 |
| Cuijpers, 2014 [24]  Palmer, 2013 [37] | CKD | All-cause mortality | 12 | 2  (6.5–1) | CBCD, SI, VSI | 922/930 | 1.34  (1.1–1.64) | 1.66  (1.2–2.3) | 0.98  (0.72–1.34) | 0.004 | 0.002 | 0.84–1.41 |
| Cuijpers, 2014 [24] | COPD | All-cause mortality | 5 | 1  (5.3–1) | CBCD, VSI | 338/261 | 2.34  (1.69–3.24) | 2.34  (1.69–3.24) | 1.93 (1.04–3.58) | < 10^-6^ | < 10^-6^ | 1.23–3.63 |
| Cuijpers, 2014 [24] Satin, 2009 [38] | CA | All-cause mortality | 23 | 4.8  (14–0.5) | VSI, SI | 4034/4817 | 1.48  (1.39–1.57) | 1.55  (1.32–1.81) | 1.37  (1.26–1.5) | < 10^-6^ | < 10^-6^ | 0.8–2.51 |
| Cuijpers, 2014 [24] Fan, 2014 [39] Gathright 2017 [40] Sokoreli, 2016 [41]. | HF | All-cause mortality | 22 | 2.7  (30–0.5) | CBCD, VSI, SI | 3418/4345 | 1.17  (1.13–1.21) | 1.46  (1.3–1.65) | 1.33  (1.19–1.42) | < 10^-6^ | < 10^-6^ | 0.89–1.92 |
| Cuijpers, 2014 [24] | HIV | All-cause mortality | 4 | 7  (10–1.5) | VSI | 1977/1580 | 1.35  (1.18–1.54) | 1.3  (1.05–1.61) | 1.6  (1.32–1.92) | < 0.001 | 0.017 | 0.61–2.71 |
| Cuijpers, 2014 [24] Dew, 2015 [42] | Posttransplant | All-cause mortality | 6 | 6.5  (12.1–0.5) | VSI, SI | 405/433 | 1.48  (1.24–1.77) | 1.53  (1.18–1.98) | 1.66  (1.12–2.47) | < 0.001 | 0.001 | 0.71–2.65 |
| Baxter, 2011 [43] Cuijpers, 2002 [44] Cuijpers, 2014 [24]  Pederson, 2016 [45] Walker, 2015 [46] | Mixed Sample | All-cause mortality | 101 | 5  (40–0.1) | CBCD, SI, VSI | 87633/242577 | 1.25  (1.23–1.27) | 1.48  (1.39–1.58) | 1.77  (1.41–2.17) | < 10^-6^ | < 10^-6^ | 0.85–2.24 |
| Correll, 2017 [47] | Mixed Sample | Cardiovascular mortality | 4 | 8.5  (14.7–4) | SI | 175726/14495 | 1.34  (1.2–1.49) | 1.56  (1.08–2.24) | 1  (0.85–1.17) | < 10^-6^ | 0.018 | 0.34–6.83 |
| **Abbreviations:**  **ACS,** acute coronary syndrome; **AMI**, acute myocardial infarction; **CA**, cancer; **CABG**, coronary artery bypass grafting; **CBCD**, criteria based clinical diagnosis; **CHD**, coronary heart disease; **CKD**, chronic kidney disease; **COPD**, Chronic obstructive pulmonary disease; **CVD**, cardiovascular disease; **DM,** diabetes mellitus; **HF**, heart failure; **K**, Number of studies; **MDD**, Major depressive disorder; **NA**, not available; **RR**, risk ratio; **SI**, structured interview; **VSI**, validated screening instrument.  ^α^ Follow-up in years.  ^‡^ Fixed effects refer to summary effect size (95% CI) using the meta-analysis fixed-effects model.  ^§^ Random effects refers to summary effect size (95% CI) using the meta-analysis random-effects model.  ^¶^ P-value of summary fixed effects estimate.  ^†^ P-value of summary random effects estimate.  ^*^ Kidney, liver, heart and lung transplantation; ^**^ Include community samples, inpatients, outpatients and primary care; ^***^Includes community samples, inpatients and outpatients | | | | | | | | | | | | |

**Table S3. Adjustment of associations of mortality and MDD in each individual study included in the umbrella review.** Each type of covariate is marked with X if included in the adjustment.

| **Association** | **Study** | **Adjusted by** | | | | | | |
| --- | --- | --- | --- | --- | --- | --- | --- | --- |
|  |  | **Age** | **Gender** | **Demographics** | **Factors related to the disease** | **Comorbidities** | **Etiology of the condition** | **Treatment** |
| DM/all-cause mortality | Bruce, 2005 | x | x | x | x | x | x | x |
|  | Egede, 2005 | x | x | x | x | x |  | x |
|  | Iversen, 2012 | x | x | x | x | x |  | x |
|  | Lin, 2009 |  |  |  |  |  |  |  |
|  | Richardson, 2008 | x |  | x |  | x |  |  |
|  | Rosenthal, 1988 | x |  |  | x | x |  |  |
|  | Ting , 2013 | x | x |  | x | x | x | x |
|  | Winkley, 2012 | x | x | x | x | x |  |  |
|  | Zhang , 2005 | x | x | x |  |  |  |  |
| DM/cardiovascular mortality | Lin, 2009 | x | x | x | x | x |  | x |
|  | Bruce, 2005 | x | x | x | x | x | x | x |
|  | Egede, 2005 | x | x | x | x | x |  | x |
| Post AMI/all-cause mortality | Carney, 2009 | x |  |  | x | x |  | x |
|  | Frassure-Smith, 1993 |  |  | x |  |  |  |  |
|  | Frassure-Smith, 1995 |  |  | x |  |  |  |  |
|  | Lauzon, 2003 | x | x |  | x | x |  |  |
|  | Rumsfeld, 2005 | x |  |  | x |  |  |  |
|  | Sorensen, 1997 | x |  | x | x |  |  |  |
|  | Welin, 2000 |  | x |  | x |  |  |  |
|  | Wheeler, 2012 | x |  |  |  |  |  |  |
|  | Zuidersma, 2013 | x | x |  | x | x |  |  |
| Post AMI/ cardiovascular mortality | Frasure-Smith, 1999 | x | x |  | x | x |  |  |
|  | Lane, 2001 | x | x | x |  | x |  |  |
|  | Welin, 2000 | x | x | x | x | x |  |  |
| Stroke/all-cause mortality | Ellis, 2010 | x | x | x | x | x |  | x |
|  | Melkas, 2010 | x | x | x | x | x |  |  |
|  | Williams, 2004 | x | x | x | x | x |  |  |

| Stroke | Everson, 1998 | x | x | x |  |  |  |  |
| --- | --- | --- | --- | --- | --- | --- | --- | --- |
|  | Surtees, 2008 | x | x | x |  | x |  | x |
|  | Yasuda, 2002 | x | x | x | x | x |  |  |
| ACS/all-cause mortality | Kronish, 2009 |  |  | x | x |  |  |  |
| CHD/all-cause mortality | Bosworth, 1999 |  |  | x | x | x |  |  |
|  | Herrmann, 2000 | x | x |  | x | x |  |  |
| CABG/all-cause mortality | Blumenthal, 2003 | x | x |  | x | x |  |  |
|  | Connerney, 2010 | x | x |  | x | x |  |  |
|  | Tully, 2008 | x |  |  | x | x |  |  |
| CKD/all-cause mortality | Balogun, 2006 | x |  |  |  |  |  |  |
|  | Chilcot, 2011 |  |  |  | x | x |  |  |
|  | Diefenthaeler, 2008 | x |  |  | x | x |  |  |
|  | Fischer, 2011 | x | x |  | x | x |  |  |
|  | Hedayati, 2010 | x |  | x |  |  |  |  |
|  | Hedayati, 2005 | x |  | x |  | x | x |  |
|  | Kojima, 2010 | x | x | x | x | x |  |  |
|  | Riezebos, 2010 | x | x |  | x | x |  |  |
| COPD/all-cause mortality | Almagro, 2002 |  |  | x | x | x |  |  |
|  | De Voogd, 2009 | x | x | x | x | x | x |  |
|  | Ng, 2007 | x |  | x | x | x | x |  |
|  | Papaiounaou, 2013 | x | x |  | x | x | x |  |
|  | Yohannes, 2005 |  |  |  |  |  |  |  |
| CA/all-cause mortality | Arrieta, 2012 | x | x |  | x | x | x |  |
|  | Chen, 2011 | x |  |  | x |  |  |  |
|  | Cohen, 2012 |  |  | x | x |  |  |  |
|  | Gripp, 2007 |  |  |  | x | x | x | x |
|  | Groenvold, 2007 | x |  | x | x | x |  | x |
|  | Karvonen-Gutierrez , 2008 | x |  | x | x | x | x |  |
|  | Nakaya, 2006 | x | x |  | x | x | x |  |
|  | Nakaya, 2008 | x | x | x | x | x | x |  |
|  | Pirl, 2008 |  |  |  | x | x |  |  |
|  | Prieto, 2005 | x |  |  | x |  | x |  |
|  | Steel, 2007 | x | x | x | x | x | x |  |
|  | Stommel, 2002 | x | x | x | x | x | x |  |
|  | Suthahar, 2008 | x | x | x | x |  | x |  |
|  | Tian , 2009 | x | x |  | x |  |  |  |
| HF/all-cause mortality | Adams, 2012 | x |  | x | x | x | x |  |
|  | Alhurani, 2015 | x | x | x | x | x |  |  |
|  | Chung , 2009 | x | x | x | x |  |  |  |
|  | Diez-Quevedo, 2013 | x | x |  | x | x | x | x |
|  | Faller, 2007 | x | x | x | x |  | x |  |
|  | Freedland, 1991 |  |  |  |  |  |  |  |
|  | Hedayati, 2004 | x |  |  |  | x |  |  |
|  | Jiang, 2007 | x |  | x | x | x | x |  |
|  | Junger, 2005 |  |  |  | x |  |  |  |
|  | Kato, 2009 | x |  |  | x |  |  | x |
|  | Lesman-Leegte, 2009 | x | x |  | x |  |  |  |
|  | Mommersteeg, 2016 | x | x |  |  | x | x |  |
|  | Moraska, 2013 | x | x |  | x | x |  |  |
|  | O'Connor, 2008 | x | x | x | x |  | x |  |
|  | Rollman, 2012 | x | x |  | x | x |  | x |
|  | Sherwood, 2007 | x |  |  | x |  | x |  |
|  | Testa, 2011 | x | x |  |  | x |  |  |
|  | van den Broek, 2011 | x | x | x | x | x | x |  |
|  | Volz, 2011 |  |  |  | x |  |  |  |
|  | Zuluaga, 2010 | x | x | x | x | x | x | x |
| HIV/all-cause mortality | French, 2009 | x | x | x | x | x |  | x |
|  | Lyketsos, 1996 | x | x | x | x |  |  | x |
|  | Page-Shafer, 1996 | x | x |  | x | x |  |  |
| Posttransplant/all-cause mortality | Novak, 2010 | x | x |  | x | x |  |  |
|  | Rosenberg, 2016 | x |  |  | x | x |  | x |
| Mixed samples/all-cause mortality | Aaroma, 1994 | x |  | x | x | x | x |  |
|  | Abbatecola, 2011 | x | x | x |  |  |  |  |
|  | Adamson, 2005 | x | x |  |  | x |  |  |
|  | Almeida, 2010 | x | x |  |  |  | x |  |
|  | Amador, 2006 | x | x |  | x | x |  |  |
|  | Anstey, 2002 | x | x | x |  | x |  |  |
|  | Arfken, 1999 | x | x | x | x | x |  |  |
|  | Atlantis, 2011 | x | x | x | x | x |  |  |
|  | Ben-Ezra, 2006 | x | x | x | x | x |  |  |
|  | Black, 1999 | x | x | x |  | x |  |  |
|  | Bosworth, 1999 | x | x | x | x | x |  |  |
|  | Bot, 2012 | x | x |  | x | x |  |  |
|  | Brill, 1992 |  |  |  | x |  |  |  |
|  | Bula, 2001 | x | x | x | x | x |  |  |
|  | Callahan, 1998 | x | x | x | x | x |  |  |
|  | Chwastiak, 2010 | x | x | x |  |  |  |  |
|  | Clausen,, 2007 | x |  |  |  |  |  |  |
|  | Covinsky, 1999 | x | x | x |  | x |  |  |
|  | Drago, 2007 | x | x | x | x | x | x | x |
|  | Eaton, 2013 | x | x | x |  |  |  |  |
|  | Engedal, 1996 |  | x | x |  | x |  |  |
|  | Fortes, 2011 | x | x | x |  |  |  |  |
|  | Fu, 2003 | x | x | x | x |  |  |  |
|  | Gale, 2012 | x |  | x |  | x |  |  |
|  | Gallo, 2005 |  |  |  | x | x |  |  |
|  | Ganzini, 1997 |  |  | x | x |  |  |  |
|  | Guerini, 2010 | x | x |  | x | x |  |  |
|  | Hamer, 2011 | x | x | x | x | x |  | x |
|  | Helmer , 1999 |  |  | x | X | x |  |  |
|  | Herrmann, 1998 |  |  | x |  | x |  |  |
|  | Herrmann-Lingen, 2001 |  |  |  | X | x |  |  |
|  | Hjaltadottir, 2011 |  |  |  |  |  |  |  |
|  | Ho, 2005 | x |  |  | X | x |  |  |
|  | Imai , 2012 | x | x |  | X |  |  |  |
|  | Janzing , 1999 | x | x |  | X | x |  |  |
|  | Kerr, 2011 |  |  | x | X | x |  |  |
|  | Kinder, 2008 | x |  | x | X | x |  |  |
|  | Koenig, 1989 | x |  |  | X | x |  |  |
|  | Kohler, 2013 | x |  | x | X | x |  |  |
|  | Kojima, 2010 | x |  |  |  |  |  |  |
|  | Kopp (male), 2011 | x |  |  | X |  |  |  |
|  | Krause, 2008 |  |  | x | X |  |  |  |
|  | Kuzuya, 2006 | x |  | x | X | x |  | x |
|  | Laan, 2011 | x |  | x |  |  |  |  |
|  | Lawrence, 2000 | x |  | x | X |  |  |  |
|  | Lemogne , 2012 | x |  | x | X | x |  |  |
|  | Mallon, 2002 | x |  |  |  | x |  | x |
|  | Markkula, 2012 |  |  | x | X | x |  |  |
|  | Marzari , 2005 |  |  |  |  | x |  |  |
|  | McCusker, 2006 |  |  | x |  | x |  |  |
|  | Meller, 1999 | x |  | x |  |  |  |  |
|  | Mogga, 2006 | x | x |  |  |  |  |  |
|  | Mykletun, 2009 | x | x | x |  | x |  |  |
|  | Nabi, 2010 | x | x |  |  |  |  |  |
|  | Onitilo, 2006 | x | x | x | X | x |  | x |
|  | Patten, 2011 | x | x | x |  | x |  |  |
|  | Penninx, 1998 | x | x |  | X | x |  |  |
|  | Phillips, 2009 |  |  |  |  |  |  |  |
|  | Pieper, 2011 | x | x |  | X | x |  |  |
|  | Pollak , 1990 | x |  | x |  | x |  |  |
|  | Riezebos, 2010 | x | x |  | X | x |  |  |
|  | Ryan , 2008 |  |  | x | X | x |  |  |
|  | Saz, 1999 | x | x | x |  |  |  |  |
|  | Schoevers , 2000 | x |  | x | X | x |  |  |
|  | Schulz, 2000 | x | x | x | X |  |  |  |
|  | Sharifi, 2012 | x | x | x | X | x | x |  |
|  | St John, 2012 | x | x | x |  |  |  |  |
|  | Whooley, 1998 | x | x |  | X | x |  |  |
|  | Wulsin, 2005 | x | x |  |  | x |  |  |
|  | Wyman, 2012 | x | x |  |  |  |  |  |
|  | Yaffe, 2003 | x | x | x |  |  |  |  |
|  | Young, 2010 | x | x | x | X | x |  |  |
|  | Zhang , 2005 | x | x | x |  |  |  |  |
| Mixed samples/cardiovascular mortality | Almeida , 2014 | x |  | x | X | x |  |  |
|  | Murray Thomas, 2013 | x | x |  | X |  |  |  |
|  | Saint Onge, 2014 | x | x | x |  |  |  |  |

**Abbreviations:** **NA,** not adjusted; **NI**, adjustment covariates not informed.

**Table S4. AMSTAR rating of the included systematic reviews and meta-analyses.**

| **Reference** | **AMSTAR Item** | | | | | | | | | | | **Total** |
| --- | --- | --- | --- | --- | --- | --- | --- | --- | --- | --- | --- | --- |
|  | **1** | **2** | **3** | **4** | **5** | **6** | **7** | **8** | **9** | **10** | **11** |  |
| Barber, 2016 [48] | CA | Y | Y | Y | N | Y | Y | Y | N | N | Y | **7** |
| Barth, 2004 [34] | CA | N | Y | N | Y | Y | N | N | Y | Y | Y | **6** |
| Bartoli, 2013 [32] | CA | N | N | N | N | Y | N | N | Y | Y | Y | **4** |
| Baxter, 2011 [43] | CA | N | Y | Y | N | Y | N | N | Y | Y | N | **5** |
| Correll, 2017 [47] | Y | Y | Y | N | N | Y | Y | Y | Y | Y | N | **8** |
| Cuijpers, 2002 [44] | CA | CA | Y | N | N | Y | N | N | Y | N | N | **3** |
| Cuijpers, 2013 [49] | CA | CA | Y | N | N | N | Y | N | Y | Y | Y | **5** |
| Cuijpers, 2014 [24] | CA | CA | Y | N | N | Y | Y | Y | Y | Y | Y | **7** |
| Dew, 2015 [42] | Y | Y | Y | N | N | Y | Y | Y | Y | Y | Y | **9** |
| van Dooren, 2013 [27] | CA | Y | Y | N | N | Y | N | N | Y | Y | Y | **6** |
| Fan, 2014 [39] | CA | N | Y | N | N | Y | Y | Y | Y | Y | Y | **7** |
| Gathright 2017 [40] | N | CA | Y | CA | N | Y | N | N | Y | Y | Y | **5** |
| Hofmann, 2013 [25] | CA | Y | Y | N | Y | Y | Y | Y | Y | Y | Y | **9** |
| Leung, 2012 [35] | CA | N | Y | N | N | Y | Y | Y | Y | Y | Y | **7** |
| Meijer, 2011 [31] | CA | CA | Y | CA | N | Y | Y | N | Y | Y | N | **5** |
| Meijer, 2013 [50] | N | Y | Y | N | N | Y | N | N | N | N | Y | **4** |
| van Melle, 2004 [51] | CA | N | Y | N | N | Y | N | N | Y | Y | N | **4** |
| Palmer, 2013 [37] | CA | N | N | N | Y | Y | Y | Y | Y | Y | Y | **7** |
| Pan, 2011 [33] | CA | Y | Y | N | N | Y | Y | Y | Y | Y | Y | **8** |
| Park, 2013 [26] | CA | Y | Y | N | Y | Y | Y | Y | Y | Y | Y | **9** |
| Pederson, 2016 [45] | CA | Y | Y | N | N | Y | Y | Y | Y | Y | Y | **8** |
| Salte, 2015 [52] | CA | N | Y | N | N | Y | Y | N | N | N | Y | **4** |
| Satin, 2009 [38] | CA | N | Y | N | N | Y | N | N | Y | Y | Y | **5** |
| Sokoreli, 2016 [41] | CA | Y | Y | CA | N | Y | N | N | Y | N | Y | **7** |
| Sorensen, 2005 [53] | CA | N | Y | CA | CA | Y | Y | Y | NA | NA | N | **4** |
| Stenman, 2016 [36] | Y | N | Y | Y | N | Y | Y | Y | Y | Y | Y | **9** |
| Walker, 2015 [46] | CA | Y | Y | N | N | Y | N | N | Y | Y | Y | **6** |

| **Table S5.** **Evaluation of heterogeneity, small-study effects and excess of significance bias in 21 meta-analytic estimates investigating the associations of depression and mortality.** | | | | | | | | |
| --- | --- | --- | --- | --- | --- | --- | --- | --- |
| **Author, year** | **Population** | **Mortality type** | **Egger's P-value**^¶, †^ | **I^2^ / P**^‡^ | **Number of studies** | **Excess of significance** | | |
|  |  |  |  |  |  | **Observed** | **Expected** | **P-value** |
| Cuijpers, 2014 [24] Hofmann, 2013 [25] Park, 2013 [26] | DM | All-cause mortality | 0.589 | 83.616/<0.001 | 12 | 5.726 | 1.727 | 0.189 |
| Van Dooren, 2013 [27] | DM | Cardiovascular mortality | 0.647 | 52.512/0.097 | 4 | 0.388 | 7.404 | 0.007 |
| Cuijpers, 2014 [24]  Sorensen, 2005 [28]  Van Melle, 2004 [29]  Meijer, 2013 [30] | Post AMI | All-cause mortality | 0.404 | 66.6/<0.001 | 20 | 10.969 | 0.215 | 0.643 |
| Meijer, 2011 [31] | Post AMI | Cardiovascular mortality | 0.557 | 42.353/0.139 | 5 | 2.373 | 0.316 | 0.574 |
| Bartoli, 2013 [32] Cuijpers, 2014 [24 ] Pan, 2011 [33] | Stroke | All-cause mortality | 0.014 | 62.177/0.015 | 7 | 0.05 | 316.281 | 0 |
| Pan, 2011 [33] | Stroke | Fatal Stroke | 0.758 | 46.665/0.131 | 4 | 0.018 | 221.216 | 0 |
| Cuijpers, 2014 [24]  Meijer, 2013 [30] | ACS | All-cause mortality | 0.108 | 86.386/0.001 | 3 | 0.544 | 5.357 | 0.021 |
| Barth, 2004 [34]  Cuijpers, 2014 [24]  Leung, 2012 [35]  Meijer, 2013 [30] | CHD | All-cause mortality | 0.047 | 62.637/0.004 | 10 | 1.181 | 14.22 | 0 |
| Cuijpers, 2014 [24]  Stenman, 2016 [36] | CABG | All-cause mortality | 0.966 | 0/0.766 | 4 | 2.32 | 0.105 | 0.746 |
| Cuijpers, 2014 [24]  Palmer, 2013 [37] | CKD | All-cause mortality | 0.002 | 44.369/0.049 | 12 | 0.174 | 85.532 | 0 |
| Cuijpers, 2014 [24] | COPD | All-cause mortality | 0.026 | 0/0.635 | 5 | 3.011 | 1.314 | 0.252 |
| Cuijpers, 2014 [24] Satin, 2009 [38] | CA | All-cause mortality | 0.364 | 69.597/<0.001 | 23 | 4.534 | 11.617 | 0.001 |
| Cuijpers, 2014 [24] Fan, 2014 [39] Gathright 2017 [40] Sokoreli, 2016 [41]. | HF | All-cause mortality | <0.001 | 78.413/<0.001 | 22 | 6.503 | 12.268 | 0 |
| Cuijpers, 2014 [24] | HIV | All-cause mortality | 0.388 | 55.156/0.082 | 4 | 1.797 | 0.643 | 0.423 |
| Cuijpers, 2014 [24] Dew, 2015 [42] | Posttransplant | All-cause mortality | 0.656 | 36.485/0.163 | 6 | 0.864 | 13.3 | 0 |
| Baxter, 2011 [43] Cuijpers, 2002 [44] Cuijpers, 2014 [24]  Pederson, 2016 [45] Walker, 2015 [46] | Mixed Sample | All-cause mortality | <0.001 | 89.276/0 | 101 | 17.832 | 97.157 | 0 |
| Correll, 2017 [47] | Mixed Sample | Cardiovascular mortality | 0.496 | 87.884/<0.001 | 4 | 1.006 | 5.282 | 0.022 |
| **Abbreviations:** **ACS,** acute coronary syndrome; **AMI**, acute myocardial infarction; **CA**, cancer; **CABG**, coronary artery bypass grafting; **CBCD**, criteria based clinical diagnosis; **CHD**, coronary heart disease; **CKD**, chronic kidney disease; **COPD**, Chronic obstructive pulmonary disease; **DM,** diabetes mellitus; **HF**, heart failure; **MDD**, major depressive disorder; **NA**, not available; **NE**, not evaluated because the number of observed studies with significant results was smaller than the expected number. | | | | | | | | |
| ^¶^ P-value from the Egger’s regression asymmetry test.  ^†^ Values in bold identify the studies with small-study effects (i.e., P < 0.1 in Egger’s test and the effect size of the largest study more conservative than the random-effects summary effect size).  ^‡^ I^2^ metric of inconsistency (95% CI) and the P-value of the Q test.  ^*^ Kidney, liver, heart and lung transplantation  ^**^ Include community samples, inpatients, outpatients and primary care; ^***^Includes community samples, inpatients and outpatients | | | | | | | | |

| **Table S6. Sensitivity analysis using credibility ceilings for the meta-analyses investigating the associations between depression and mortality for associations that presented highly suggestive evidence criteria.** | | | | | |
| --- | --- | --- | --- | --- | --- |
| **Reference** | **Population** | **Mortality** | **10% Credibility ceiling ES (95% CI)** | **20% Credibility ceiling ES (95% CI)** | **30% Credibility ceiling ES (95% CI)** |
| Cuijpers, 2014 [24] Satin, 2009 [38] | CA | All-cause mortality | 1.164 (1.003–1.324) | 1.088 (0.897–1.279) | 1.007 (0.776–1.237) |
| Cuijpers, 2014 [24] Fan, 2014 [39] Gathright 2017 [40] Sokoreli, 2016 [41]. | HF | All-cause mortality | 1.083 (1.018–1.148) | 1.083 (0.992–1.173) | 1.079 (0.937–1.221) |
| Baxter, 2011 [43] Cuijpers, 2002 [44] Cuijpers, 2014 [24]  Pederson, 2016 [45] Walker, 2015 [46] | Mixed Sample | All-cause mortality | 1.068 (1.028–1.109) | 1.048 (0.999–1.097) | 1.031 (0.965–1.097) |
| Cuijpers, 2014 [24]  Sorensen, 2005 [28]  van Melle, 2004 [29]  Meijer, 2013 [30] | Post-AMI | All-cause mortality | 1.458 (1.066–1.851) | 1.326 (0.829–1.824) | 1.179 (0.531–1.827) |
| **Abbreviations: CI**, confidence interval; **CKD**, chronic kidney disease; **ES**, effect size.  ^*^ Kidney, liver, heart and lung transplantation  ^**^ Include community samples, inpatients, outpatients and primary care | | | | | |

**Supplementary References**

1. Ayerbe L, Ayis S, Wolfe CD, Rudd AG: **Natural history, predictors and outcomes of depression after stroke: systematic review and meta-analysis**. *The British journal of psychiatry : the journal of mental science* 2013, **202**(1):14-21.

2. Ayerbe L, Ayis S, Wolfe CDA, Rudd AG: **Natural history, predictors and outcomes of depression after stroke: Systematic review and meta-analysis**. *British Journal of Psychiatry* 2013, **202**(1):14-21.

3. Bartoli F, Lillia N, Lax A, Crocamo C, Mantero V, Pini E, Carra G, Agostoni E, Clerici M: **The association between post-stroke depression and mortality: A metaanalysis**. *European Psychiatry Conference: 21st European Congress of Psychiatry, EPA* 2013, **28**(no pagination).

4. Bartoli F, Paolucci S: **Association of depression and SSRIs with mortality after stroke**. *Neurology* 2014, **83**(22):1998-1999.

5. Charlson FJ, Moran AE, Freedman G, Norman RE, Stapelberg NJ, Baxter AJ, Vos T, Whiteford HA: **The contribution of major depression to the global burden of ischemic heart disease: a comparative risk assessment**. *BMC medicine* 2013, **11**:250.

6. Courtwright AM, Salomon S, Lehmann LS, Wolfe DJ, Goldberg HJ: **The Effect of Pretransplant Depression and Anxiety on Survival Following Lung Transplant: A Meta-analysis**. *Psychosomatics* 2016, **57**(3):238-245.

7. Cole MG: **Does depression in older medical inpatients predict mortality? A systematic review**. *General hospital psychiatry* 2007, **29**(5):425-430.

8. Doyle F, McGee H, Conroy R, Jan Conradi H, Meijer A, Steeds R, Sato H, Stewart DE, Parakh K, Carney R *et al*: **Systematic review and individual patient data meta-analysis of sex differences in depression and prognosis in persons with myocardial infarction: A MINDMAPS study**. *Psychosomatic Medicine* 2015, **77**(4):419-428.

9. Engelmann J, Manuwald U, Rubach C, Kugler J, Birkenfeld AL, Hanefeld M, Rothe U: **Determinants of mortality in patients with type 2 diabetes: a review**. *Reviews in endocrine & metabolic disorders* 2016, **17**(1):129-137.

10. Ghoneim MM, O'Hara MW: **Depression and postoperative complications: an overview**. *BMC surgery* 2016, **16**:5.

11. Lichtman JH, Froelicher ES, Blumenthal JA, Carney RM, Doering LV, Frasure-Smith N, Freedland KE, Jaffe AS, Leifheit-Limson EC, Sheps DS *et al*: **Depression as a risk factor for poor prognosis among patients with acute coronary syndrome: systematic review and recommendations: a scientific statement from the American Heart Association**. *Circulation* 2014, **129**(12):1350-1369.

12. Malberg K: **Meta analysis: Depression increases mortality risk in cancer patients. [German]**

**Metaanalyse: Depression erhoht sterberisiko bei krebspatienten**. *MMW-Fortschritte der Medizin* 2009, **151**(41):29.

13. Marzouka GR, Martinez C, Tamariz L: **Depression increases all-cause mortality in post cardiac surgery patients: A meta-analysis of prospective cohort studies**. *Circulation: Cardiovascular Quality and Outcomes Conference: Quality of Care and Outcomes Research in Cardiovascular Disease and Stroke* 2010, **4**(6 MeetingAbstracts2010).

14. Mitchell A, Nanni MG, Grassi L: **Elevated cancer mortality in unipolar and bipolar affective disorders: A meta-analysis of population studies**. *Psycho-Oncology* 2016, **25**:116-117.

15. Park M, Katon W: **A meta-analysis of mortality rates associated with comorbid depression among older adults with diabetes**. *Journal of the American Geriatrics Society* 2012, **60**:S87.

16. Pinquart M, Duberstein PR: **Depression and cancer mortality: a meta-analysis**. *Psychological medicine* 2010, **40**(11):1797-1810.

17. Shi S, Liu T, Liang J, Hu D, Yang B: **The relationship between depression and risk of sudden cardiac death and arrhythmias: A meta-analysis**. *Heart Rhythm* 2016, **1)**:S502-S503.

18. van den Akker M, Schuurman AG, Ensinck KTJL, Buntinx F: **Depression as a risk factor for total mortality in the community: A meta-analysis**. *Archives of Public Health* 2003, **61**(6):313-332.

19. Wu Q, Kling JM: **Depression and the Risk of Myocardial Infarction and Coronary Death: A Meta-Analysis of Prospective Cohort Studies**. *Medicine* 2016, **95**(6):e2815.

20. Wyman L: **The association between depression and mortality in the general community**. *Dissertation Abstracts International: Section B: The Sciences and Engineering* 2013, **73**(12-B(E)):No Pagination Specified.

21. Maslej MM, Bolker BM, Russell MJ, Eaton K, Durisko Z, Hollon SD, Swanson GM, Thomson JA, Jr., Mulsant BH, Andrews PW: **The Mortality and Myocardial Effects of Antidepressants Are Moderated by Preexisting Cardiovascular Disease: A Meta-Analysis**. *Psychotherapy and psychosomatics* 2017, **86**(5):268-282.

22. Liu Y, Wang Z, Xiao W: **Risk factors for mortality in elderly patients with hip fractures: a meta-analysis of 18 studies**. *Aging clinical and experimental research* 2017.

23. Aitken SJ, Blyth FM, Naganathan V: **Incidence, prognostic factors and impact of postoperative delirium after major vascular surgery: A meta-analysis and systematic review**. *Vascular medicine (London, England)* 2017, **22**(5):387-397.

24. Cuijpers P, Vogelzangs N, Twisk J, Kleiboer A, Li J, Penninx BW: **Comprehensive meta-analysis of excess mortality in depression in the general community versus patients with specific illnesses**. *The American journal of psychiatry* 2014, **171**(4):453-462.

25. Hofmann M, Kohler B, Leichsenring F, Kruse J: **Depression as a risk factor for mortality in individuals with diabetes: a meta-analysis of prospective studies**. *PloS one* 2013, **8**(11):e79809.

26. Park M, Katon WJ, Wolf FM: **Depression and risk of mortality in individuals with diabetes: a meta-analysis and systematic review**. *General hospital psychiatry* 2013, **35**(3):217-225.

27. van Dooren FE, Nefs G, Schram MT, Verhey FR, Denollet J, Pouwer F: **Depression and risk of mortality in people with diabetes mellitus: a systematic review and meta-analysis**. *PloS one* 2013, **8**(3):e57058.

28. Sorensenf C, Friis-Hasche E, Haghfelt T, Bech P: **Postmyocardial infarction mortality in relation to depression: a systematic critical review**. *Psychotherapy and psychosomatics* 2005, **74**(2):69-80.

29. van Melle JP, de Jonge P, Spijkerman TA, Tijssen JG, Ormel J, van Veldhuisen DJ, van den Brink RH, van den Berg MP: **Prognostic association of depression following myocardial infarction with mortality and cardiovascular events: a meta-analysis**. *Psychosomatic medicine* 2004, **66**(6):814-822.

30. Meijer A, Conradi HJ, Bos EH, Anselmino M, Carney RM, Denollet J, Doyle F, Freedland KE, Grace SL, Hosseini SH *et al*: **Adjusted prognostic association of depression following myocardial infarction with mortality and cardiovascular events: individual patient data meta-analysis**. *The British journal of psychiatry : the journal of mental science* 2013, **203**(2):90-102.

31. Meijer A, Conradi HJ, Bos EH, Thombs BD, van Melle JP, de Jonge P: **Prognostic association of depression following myocardial infarction with mortality and cardiovascular events: a meta-analysis of 25 years of research**. *General hospital psychiatry* 2011, **33**(3):203-216.

32. Bartoli F, Lillia N, Lax A, Crocamo C, Mantero V, Carra G, Agostoni E, Clerici M: **Depression after stroke and risk of mortality: a systematic review and meta-analysis**. *Stroke research and treatment* 2013, **2013**:862978.

33. Pan A: **Depression and risk of stroke morbidity and mortality: A meta-analysis and systematic review (JAMA - Journal of the American Medical Association (2011) 306, 11 (1241-1249))**. *JAMA - Journal of the American Medical Association* 2011, **306**(23):2565.

34. Barth J, Schumacher M, Herrmann-Lingen C: **Depression as a risk factor for mortality in patients with coronary heart disease: a meta-analysis**. *Psychosomatic medicine* 2004, **66**(6):802-813.

35. Leung YW, Flora DB, Gravely S, Irvine J, Carney RM, Grace SL: **The impact of premorbid and postmorbid depression onset on mortality and cardiac morbidity among patients with coronary heart disease: meta-analysis**. *Psychosomatic medicine* 2012, **74**(8):786-801.

36. Stenman M, Holzmann MJ, Sartipy U: **Association between preoperative depression and long-term survival following coronary artery bypass surgery - A systematic review and meta-analysis**. *International journal of cardiology* 2016, **222**:462-466.

37. Palmer SC, Vecchio M, Craig JC, Tonelli M, Johnson DW, Nicolucci A, Pellegrini F, Saglimbene V, Logroscino G, Hedayati SS *et al*: **Association between depression and death in people with CKD: a meta-analysis of cohort studies**. *American journal of kidney diseases : the official journal of the National Kidney Foundation* 2013, **62**(3):493-505.

38. Satin JR, Linden W, Phillips MJ: **Depression as a predictor of disease progression and mortality in cancer patients: a meta-analysis**. *Cancer* 2009, **115**(22):5349-5361.

39. Fan H, Yu W, Zhang Q, Cao H, Li J, Wang J, Shao Y, Hu X: **Depression after heart failure and risk of cardiovascular and all-cause mortality: a meta-analysis**. *Preventive medicine* 2014, **63**:36-42.

40. Gathright EC, Goldstein CM, Josephson RA, Hughes JW: **Depression increases the risk of mortality in patients with heart failure: A meta-analysis**. *Journal of Psychosomatic Research* 2017, **94**:82-89.

41. Sokoreli I, de Vries JJG, Pauws SC, Steyerberg EW: **Depression and anxiety as predictors of mortality among heart failure patients: systematic review and meta-analysis**. *Heart Failure Reviews* 2016, **21**(1):49-63.

42. Dew MA, Rosenberger EM, Myaskovsky L, DiMartini AF, DeVito Dabbs AJ, Posluszny DM, Steel J, Switzer GE, Shellmer DA, Greenhouse JB: **Depression and Anxiety as Risk Factors for Morbidity and Mortality After Organ Transplantation: A Systematic Review and Meta-Analysis**. *Transplantation* 2015, **100**(5):988-1003.

43. Baxter AJ, Page A, Whiteford HA: **Factors influencing risk of premature mortality in community cases of depression: A meta-analytic review**. *Epidemiology Research International* 2011, **2011 (no pagination)**(832945).

44. Cuijpers P, Smit F: **Excess mortality in depression: a meta-analysis of community studies**. *Journal of affective disorders* 2002, **72**(3):227-236.

45. Pederson JL, Warkentin LM, Majumdar SR, McAlister FA: **Depressive symptoms are associated with higher rates of readmission or mortality after medical hospitalization: A systematic review and meta-analysis**. *Journal of hospital medicine* 2016, **11**(5):373-380.

46. Walker ER, McGee RE, Druss BG: **Mortality in mental disorders and global disease burden implications: a systematic review and meta-analysis**. *JAMA psychiatry* 2015, **72**(4):334-341.

47. Correll CU, Solmi M, Veronese N, Bortolato B, Rosson S, Santonastaso P, Thapa-Chhetri N, Fornaro M, Gallicchio D, Collantoni E *et al*: **Prevalence, incidence and mortality from cardiovascular disease in patients with pooled and specific severe mental illness: a large-scale meta-analysis of 3,211,768 patients and 113,383,368 controls**. *World psychiatry : official journal of the World Psychiatric Association (WPA)* 2017, **16**(2):163-180.

48. Barber B, Dergousoff J, Slater L, Harris J, O'Connell D, El-Hakim H, Biron VL, Mitchell N, Seikaly H: **Depression and Survival in Patients With Head and Neck Cancer: A Systematic Review**. *JAMA otolaryngology-- head & neck surgery* 2016, **142**(3):284-288.

49. Cuijpers P, Vogelzangs N, Twisk J, Kleiboer A, Li J, Penninx BW: **Differential mortality rates in major and subthreshold depression: meta-analysis of studies that measured both**. *The British journal of psychiatry : the journal of mental science* 2013, **202**(1):22-27.

50. Meijer A, Conradi H, Bos E, Anselmino M, Carney R, Denollet J, Doyle F, Freedland K, Grace S, Hosseini S *et al*: **Adjusted prognostic association of depression following myocardial infarction with mortality and cardiovascular events: Individual patient data meta-analysis**. *The British Journal of Psychiatry* 2013, **203**(2):90-102.

51. Van Melle JP, De Jonge P, Spijkerman TA, Tijssen JGP, Ormel J, Van Veldhuisen DJ, Van Den Brink RHS, Van Den Berg MP: **Prognostic association of depression following myocardial infarction with mortality and cardiovascular events: A meta-analysis**. *Psychosomatic medicine* 2004, **66**(6):814-822.

52. Salte K, Titlestad I, Halling A: **Depression is associated with poor prognosis in patients with chronic obstructive pulmonary disease - a systematic review**. *Danish medical journal* 2015, **62**(10):A5137.

53. Sorensen C, Friis-Hasche E, Haghfelt T, Bech P: **Postmyocardial infarction mortality in relation to depression: A systematic critical review**. *Psychotherapy and psychosomatics* 2005, **74**(2):69-80.
